# Metabolomic and volatile profiling reveals defence-related effects of *Ib*Pep1 in sweet potato cell culture

**DOI:** 10.1186/s12870-026-08547-1

**Published:** 2026-03-12

**Authors:** Liza Zhyr, Christianne Mae Dela Cruz, Axel Mithöfer

**Affiliations:** 1 Research Group Plant Defense Physiology, Max Planck Institute for Chemical Ecology, Jena, 07745 Germany; 2 Flora Biodiversity Laboratory, Premier Research Institute of Science and Mathematics (PRISM), Mindanao State University - Iligan Institute of Technology, Iligan, 9200 Philippines

**Keywords:** *Ipomoea batatas*, Plant peptide elicitors, Cell suspension culture, Metabolomics, Volatiles

## Abstract

**Background:**

Damage-associated molecular patterns (DAMPs), which are recognised by specific plant receptors and trigger downstream signaling cascades that control various functions within plants, have recently emerged as key regulators of plant physiology. Over the years, researchers have identified novel peptides like systemin and HypSys to play crucial roles in defense, and their thorough characterisation has highlighted their biotechnological potential. Plants, being repositories of such compounds, encourage researchers to explore this seemingly limitless potential. Thus, in this study, we investigated *Ib*Pep1, an elicitor peptide identified in sweet potato (*Ipomoea batatas*), to assess its effects on plant metabolism and its potential role in defence responses.

**Results:**

We generated a sweet potato cell suspension culture and analysed its response to the recently discovered plant elicitor peptide, *Ib*Pep1 in comparison with other known elicitors, including flg22. In contrast to the latter, *Ib*Pep1 did not elicit a significant ROS burst but at higher concentration peptide treatment significantly increased JA-Ile concentration. The levels of free amino acids remained unaffected. Strikingly, untargeted metabolomics revealed that *Ib*Pep1 mostly affected the shikimate and phenylpropanoid pathways and volatiles’ analysis showed a stimulated emission of sesquiterpenes by *Ib*Pep.

**Conclusions:**

The structural diversity and incompatibility between families make the study of Peps challenging. However, due to their widespread presence in angiosperms, they are attractive targets for biotechnological applications. In our study, we compared the effects of *Ib*Pep1 with those of known elicitors and discovered that its regulatory functions share some similarities with signaling peptides from other plant families, while also revealing novel effects such as the regulation of volatile compounds. These results shed light on the signaling activity of *Ib*Pep1 and its impact on specialised metabolic pathways, emphasising its potential role in plant defence and metabolic regulation.

**Supplementary Information:**

The online version contains supplementary material available at 10.1186/s12870-026-08547-1.

## Introduction

Plants use a variety of signaling compounds to manage their response to environmental changes. Some of these signals originate within the plant itself and are therefore termed endogenous. Examples include compounds of various origins released as a result of damage. They are known as damage-associated molecular patterns (DAMPs) [1]. Exogenous signaling compounds from outside the plant organism may have a microbial origin and are therefore called microbial-associated molecular patterns, or MAMPs [2]. DAMPs and MAMPs are recognized by pattern-recognition receptors (PRRs), which activate pattern-triggered immunity (PTI) [3]. Depending on whether DAMPs are present under non-stressed conditions or produced in response to stress, they are classified as constitutive or inducible, respectively. Constitutive DAMPs are normally present in unstressed plants but can trigger defence responses when perceived outside their native location (e.g., cell wall fragments in the apoplast). Inducible DAMPs, on the other hand, occur upon stress and are sometimes referred to as phytocytokines [4]. Short peptide molecules that are often cleaved from inactive precursors fall into this group. These signaling peptides are now considered part of the broader class of plant hormones and include plant elicitor peptides (Peps), systemin, hydroxyproline-rich systemins (HypSys), cysteine-rich peptides, etc [5, 6]. Systemin was the first peptide hormone discovered, identified in tomato, where it is activated by wounding or herbivore attack [7]. So far, systemin signaling has been reported only in the Solanaceae family [8], whereas Peps occur across many plant species and share little sequence similarity with systemin or with each other [9, 10]. Peps are cleaved from their precursors, ProPeps, and are perceived by leucine-rich repeat receptor-like kinases known as Pep receptors (PEPRs). Despite Peps variability and interfamily incompatibility, PEPRs were found to be compatible across different plant families [10]. The presence of Peps across angiosperms makes them a valuable target for crop resistance development.

Sweet potato (*Ipomoea batatas* [L.] Lam.), the sixth most important crop worldwide, serves as an essential food source in many regions and is also widely used in animal feed and industrial applications. Increasing demand calls for new cultivars that combine high nutritional value with enhanced resistance to herbivores and pathogens. However, its heterozygous hexaploid genome makes sweet potato a particularly challenging species for traditional breeding approaches [11, 12]. *Ib*preproHypSys, the precursor of the first peptide hormone identified in sweet potato (*Ib*HypSysIV), was shown to reduce the growth of the generalist herbivore *Spodoptera littoralis* [13, 14]. This finding implies the presence of a functional peptide hormone signaling in *I. batatas*. Recently, the first plant elicitor peptide (Pep) identified in sweet potato, *Ib*Pep1, was discovered along with its compatible receptor *Ib*LRR-RK1 [15]. *Ib*Pep1 was shown to upregulate the gene responsible for sporamin production - a storage protein known for its defensive role, particularly due to its trypsin-inhibiting activity, which eventually suppresses herbivore growth [15, 16]. Nevertheless, the broader metabolic impact of *Ib*Pep1, its specific functions in plant defence, and its comparison to other known peptides and elicitors remain unexplored.

Plant cell suspension (PCS) systems have recently gained growing interest in both research and industry due to their scalability, rapid growth, and ease of genetic and biochemical manipulation. Their controlled conditions allow compliance with strict manufacturing and regulatory standards, while also providing simplified yet physiologically relevant models for biochemical and molecular biology studies [17]. Since plants are constantly exposed to diverse environmental stressors, understanding and optimising their defence mechanisms remains a central goal in plant biology. Early signaling events such as ROS generation, and later responses including stress-related phytohormone accumulation, result in global metabolic reprogramming. Investigating these processes in a versatile system like PCSs offers valuable insight into plant signaling dynamics under restricted conditions. Comparable to protoplast systems, PCSs provide a controllable and reproducible platform for studying cellular responses and signaling cascades, while also serving as a foundation for future bioengineering applications. An *I. batatas* cell culture thus represents an efficient model for the initial investigation of peptide-induced defence and metabolic changes, without the complexity of whole-plant systems [18].

In this study, we aimed to explore the metabolic and defence-related responses of *Ipomoea batatas* cell suspension culture to the plant elicitor peptide *Ib*Pep1. Our analysis combined early signaling attributes, such as reactive oxygen species (ROS) production, with later metabolic changes, including phytohormones, amino acids, global metabolite composition, and volatile emission patterns into a broader view.

## Materials and methods

### Cell culture generation and growth conditions

We used the sweet potato cultivar Tainong 57 for callus generation and propagation. We obtained plants from Prof. Dr. K-W Yeh, from National Taiwan University, Taipei, Taiwan in 2015. The leaves of young sweet potato plants were removed from the stem, which was then cut into smaller pieces (2–4 cm). The cuttings were then placed in bottles of autoclaved water. After an initial pre-wash, the water was replaced with 70% ethanol and the explants were shaken for 2 min. After removal of the ethanol, the cuttings were washed for 10 min in a solution of 0.2–0.3% sodium hypochlorite with 0.01% Tween-20 and shaken occasionally. After the solution was poured off, the explants were washed 5–7 more times with autoclaved water till no foam was formed. While nodes can be used for tissue culture, internodes cut into 0.5 cm pieces were used for callus formation. These were placed on a Petri dish containing B5 medium including vitamins (Duchefa Biochemie B.V, RV Haarlem, The Netherlands) enriched with 10 mg/L of 2,4-dichlorophenoxyacetic acid, 2% sucrose, 300 mg/L casein and 3 g/L gelrite. The plates were then transferred to a dark environment at 22 °C. After 4–6 weeks, the formed callus pieces were separated from the left stem material and transferred to fresh medium. For cell suspension culture, a piece of callus (d ~ 1–2 cm) was transferred to a flask containing mentioned above B5 media without gelrite and shaken with 115 rpm in the dark for 4 weeks. Large clumps were separated and removed by the decantation method. When homogeneity of the cell suspension was achieved, cells were subcultured every two weeks by pouring 2–3 mL of settled cells (avoiding old media) into a flask containing 40 mL of media [19]. For growth curve characterisation, we collected 0.5 mL of suspension every second day while keeping conditions completely aseptic. After the washing procedure and removal of media residues, tubes with aliquots were placed in a 60 °C oven. Cell viability was checked by adding fluorescein diacetate (FDA) or propidium iodide (PI) (Sigma-Aldrich, Taufkirchen, Germany) to discriminate living from dead cells. For treatment experiments, we used 7-day-old cells.

### Experimental design

For metabolic characterisation we quantified the phytohormones, amino acids, volatile organic compounds (VOCs) emission, and analysed metabolic profiles using untargeted metabolic approaches. For treatments, we used *Ib*Pep1 (GenScript Biotech, Leiden, Netherlands) [15], flagellin peptide flg22 (EZBiolab, Carmel, IN, USA), chitosan (Sigma-Aldrich), and cellotriose (Megazyme, Bray, Ireland). All elicitors, except chitosan, were dissolved in water. Chitosan stock solutions were prepared in a weak acetic acid solution and subsequently diluted with water to the required working concentrations. Water was used as the negative control for all analyses.

Previous studies on peptide-induced plant responses have used a wide range of elicitor concentrations; for example, studies in sweet potato applied 25 µM *Ib*Pep1 and 75 µM *Ib*HypSys [14, 15]. In the present study, lower concentrations were selected because plant cells were in direct contact with the peptide. We tested two concentrations for phytohormone and amino acid analyses (1 µM and 10 µM). In contrast, we only used the lower concentration (1 µM) for volatile analysis and untargeted metabolomics because of the extended treatment time. Concentrations of elicitors were preselected based on preliminary experiments. Treatment duration was chosen based on previous reports showing changes in phytohormones within 1–2 h after treatment [9]. While amino acid responses can occur over a broader temporal range, this early time point was selected to assess potential fast primary metabolic adjustments accompanying the hormonal response. Volatile collection required longer incubation to allow sufficient compound collection; therefore, a treatment duration of 24 h was used. Plant cells collected after 24 h were subsequently used for untargeted metabolomics to capture global metabolic reprogramming.

**Fig. 1:**
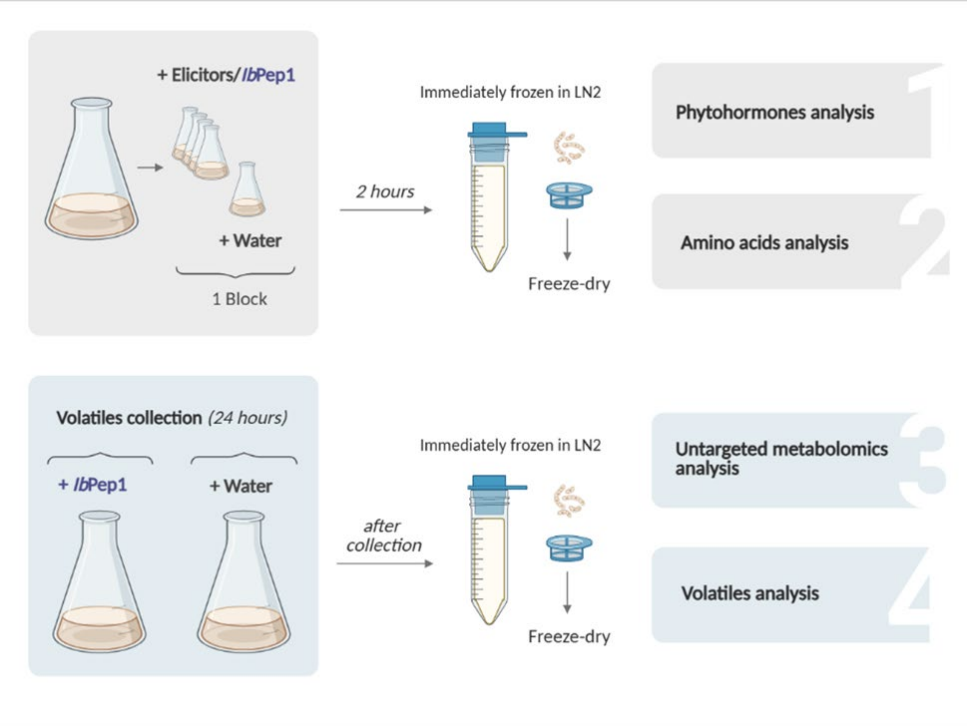
Schematic representation of the experimental design. LN2, liquid nitrogen

After treatments, fresh cells were collected either after two hours of elicitor treatment or a 24-hour period of volatile collection (Fig. 1). For further analyses, the cells were filtered through cell strainers and washed with 30 mL of MilliQ water. The filtered cells were then transferred into a tube and rapidly frozen in liquid nitrogen before being stored at -80 °C. Before analysis, cells were freeze-dried over 3 days to get dry weight (DW) material. This was conducted using the freeze-dryer Christ 1–4 LD Plus (Christ Gefriertrocknungsanlagen GmbH, Osterode am Harz, Germany).

### Reactive oxygen species quantification

The method described by Heyman et al. [20] was modified and used to quantify oxidative burst. 7-day-old cells were adjusted to a concentration of 100 mg FW/mL with fresh media, and 150 µL were added to every well. After adding the reaction mixture consisting of luminol derivative L-012 (FUJIFILM Wako Chemicals Europe GmbH, Neuss, Germany) and horseradish peroxidase (Sigma-Aldrich) in a final concentration of 30 µM and 2U, respectively, the cells were kept in a 96-well plate for 1.5 h to destress. A luminometer (Luminoskan Ascent v2.4, Thermo Fisher Scientific, Dreieich, Germany) was used for luminescence detection. Measurements were taken every min with an integration time of 1 s, with the first 10 min as a baseline. Elicitors (or water as a negative control) were applied in a final concentration of 1 µM using a multichannel pipette, and luminescence was measured for the next 50 min. Results are presented as relative light units (RLUs), where one value represents the average of 5 technical replicates (from the same cell culture batch).

### Phytohormone determination

The elicitor experiments to check stress-related phytohormones (jasmonic acid, JA; jasmonoyl-isoleucine, JA-Ile; *cis*-12-oxo-phytodienoic acid, *cis*-OPDA; salicylic acid, SA; ABA, abscisic acid) were conducted in a block-type experimental setup. The block design was implemented by dividing one flask containing a 7-day-old cell culture into seven smaller flasks (Fig. 1), each containing 10 mL of the original solution. Each small flask was treated with 0.5 mL of the elicitors, resulting in a final compound concentration of 1 or 10 µM. Subsequently, the flasks were returned to a shaker for a period of two hours. A total of 20 mg DW from each sample was extracted with 1 mL of pure methanol, which was enriched with phytohormone standards. These included 40 ng/mL of D4-SA (Santa Cruz Biotechnology, Santa Cruz, USA), 40 ng/mL of D6-JA (HPC Standards GmbH, Cunnersdorf, Germany), D6-ABA (Toronto, Research Chemicals, Toronto, Canada), and 8 ng/mL of D6-jasmonoyl-L-isoleucine (HPC Standards GmbH, Cunnersdorf, Germany). The tubes were subjected to rigorous vortexing and mixing for 30 min. The samples were subsequently subjected to centrifugation for 30 min at 16,000 rcf and at a temperature of 4 °C. The collected supernatants were used directly for the analyses. The quantification of phytohormones was conducted using an Agilent 1260 HPLC system (Agilent Technologies, Santa Clara, CA, USA) coupled to a QTRAP 6500 tandem mass spectrometer (AB Sciex, Darmstadt, Germany) equipped with a turbo spray ion source operated in negative ionisation mode as described in [21].

### Free amino acids analysis

Methanol extracts collected from the cells after 2 hours elicitors treatments were diluted 1:10 with the solution of an algal amino acid mixture (10 mg/mL U-^13^C, U-^15^N isotopically labelled amino acid mix; Isotec, Miamisburg, OH, USA) as an internal standard. The extracts treated with a 1 µM elicitor concentration were analysed on an Agilent 1260 Infinity high-performance liquid chromatography system (Agilent Technologies) coupled to an API6500 ESI-Triple Quad mass spectrometer (AB Sciex). Extracts of cells treated with 10 µM elicitors were analysed on the HPLC system HP 1260 series coupled to an API5000 tandem mass spectrometer (Applied Biosystems, Darmstadt, Germany) equipped with a Turbospray ion source operated in positive ionization mode [22]. Separation was achieved on a Zorbax Eclipse XDB-C18 column (50 × 4.6 mm, 1.8 μm, Agilent Technologies). Formic acid (0.05%) in water and acetonitrile were used as mobile phases A and B, respectively. The elution program was as follows: 0–1 min, 3% B in A; 1.0–2.7 min, 3-100% B in A; 2.7–3.0 min, 100% B in A; and 3.1–6.0 min, 3% B in A. The column temperature was maintained at 20 °C, with a constant flow rate of 1.1 mL/min. The mass spectrometer operated in positive ionization mode using multiple reaction monitoring (MRM) as described by [23]. Data acquisition and processing were performed using Analyst 1.6.3 software (Applied Biosystems). Quantification of individual amino acids was achieved using the corresponding ^13^C, ^15^N-labelled amino acid internal standards, except for tryptophan, which was quantified relative to ^13^C, ^15^N-phenylalanine with a response factor of 0.42.

### Untargeted metabolomic analysis

Flasks containing 20 mL of independently grown 7-day-old cell suspension were treated with *Ib*Pep1 (final concentration 1 µM) or water (negative control) for 24 h (Fig. 1). Freeze-dried cells were extracted with methanol as described in the “Phytohormone determination” section. Untargeted metabolomic profiling of the methanolic extracts was performed using ultra-high-performance liquid chromatography-electrospray ionisation-high resolution mass spectrometry as described by Müller et al. [21]. Samples were randomised and quality controls were measured at the beginning, middle, and end of the measurement. Peak detection was performed using the T-Rex 3D algorithm for qTOF data in MetaboScape software (Bruker Daltonik, Bremen, Germany). For peak detection, the following parameters were applied: an intensity threshold of 300, a minimum of 10 spectra, and a retention time window of 0.4–11.8 min. Peaks were retained if detected in at least 3 samples. Adducts including [M + H]⁺, [M + Na]⁺, and [M + K]⁺ (positive mode) were grouped into a single bucket when their extracted ion chromatograms (EICs) showed a correlation coefficient of ≥ 0.8. After data acquisition and peak alignment, false-positive signals were removed using the “Notame**”** workflow and package in flag detection mode [24].

To analyse metabolites obtained from the untargeted metabolomic profiling of *I. batatas* cell suspension, we used the SIRIUS (version 6.3.0) framework software with integrated CSI: FingerID [25]. The chemical taxonomy of the predicted metabolite structures, including pathway and class levels of natural product classification (NP), was determined by CANOPUS [26] using the NPClassifier chemical taxonomy [27].

### Volatile collection and analysis

Headspace volatiles from a 20 mL cell suspension were collected over 24 hours during treatment with 1 µM *Ib*Pep1 or water using a push headspace collection system in sealed 100 mL flasks. Charcoal-filtered air was pumped into a flask at a flow rate of 0.25 L/min, and excessive air was exiting through a 20 mg PoraPak filter (Alltech, Deerfield, IL, USA). The filter was trapping volatile compounds, which were eluted from them using 200 µL of dichloromethane containing 10 ng/µL *n*-bromodecane (Sigma-Aldrich) as an internal standard. Samples were stored at -20 °C prior to measurement. The analysis was performed on a Hewlett-Packard 6890 gas chromatograph (Agilent Technologies) equipped with a 30 m × 0.25 mm × 0.25 μm Optima-5 column (Macherey-Nagel, Düren, Germany) and a 1 µL splitless injector as described in [21]. Quantification was performed using a flame ionisation detector (FID) set at 300 °C. The peak areas of compounds were compared with those of the internal standard *n*-bromodecane, applying equal response factors and normalized by cell culture density harvested at the end of the collection (DW of 1 mL of suspension). A mixture of *n*-alkanes (C_8_-C_20_) dissolved in *n*-hexane (Sigma-Aldrich) was analysed before and after each sample sequence under identical GC-MS conditions. Kovats retention indices (KI) for individual chromatographic peaks were calculated based on the retention times of the *n*-alkanes using the method of van den Dool and Kratz [28]. The NIST Mass Spectral Search Program (National Institute of Standards and Technology, Gaithersburg, MD, USA) was used to search the NIST/EPA/NIH EI Mass Spectral Library (NIST 17) in order to tentatively identify the compounds. The candidates were confirmed by calculated KI (compared to those reported in NIST Chemistry WebBook and PubChem libraries) and by identified authentic standards (except τ-muurolol).

### Statistical analyses

For statistical analysis and visualization, we used R version 4.4.1 [29]. Data sets were tested for normal distribution (Shapiro-Wilk test), homogeneity of variances (Levene’s test), followed by Box-Cox transformation if the requirements for the test were not met, or in the case of amino acids analysis, we shifted to robust linear mixed-effects model. ROS measurements were analysed using a linear mixed-effects model with time, treatment, and their interaction as fixed effects, and independent replicates included as a random intercept. Pairwise comparisons between treatments within each time point were performed with Benjamini–Hochberg correction for multiple testing. Analysis of the experiments with a block design was conducted in the following way: to assess the effect of treatment on amino acids or phytohormones concentrations, we fitted a linear mixed-effects model. The model included *Treatment* as a fixed effect and *Block* as a random intercept (*model1: compound ~ treatment + (1 | block)*). We compared this model to a null model containing only the random intercept (*model2: compound ~ 1 + (1 | block)*) using a likelihood ratio test (LRT) to evaluate the significance of the treatment effect. For phytohormones, pairwise comparisons among treatments were conducted using Tukey’s HSD. For amino acids, comparisons between each elicitor treatment and the control was performed using treatment-versus-control contrasts (“trt.vs.ctrl”), implemented in the “emmeans” R-package, with multiplicity adjustment using the Dunnett-type “dunnettx” method. For volatile analysis, Student’s t-tests were used or Wilcoxon rank sum tests if data were not normally distributed. Additionally, details of statistical tests for each analysis are described in the figure legends. For untargeted metabolomics, we pre-processed data using MetaboScape (Bruker Daltonics) and then used “mixOmics” package in R for analysis [30]. Data were normalised by Pareto scaling and log-transformed. For visualisation we used “ggplot2” package [31] and “mixOmics” for untargeted metabolomics data.

## Results

### Characterisation of *I. batatas* cell suspension culture

**Fig. 2:**
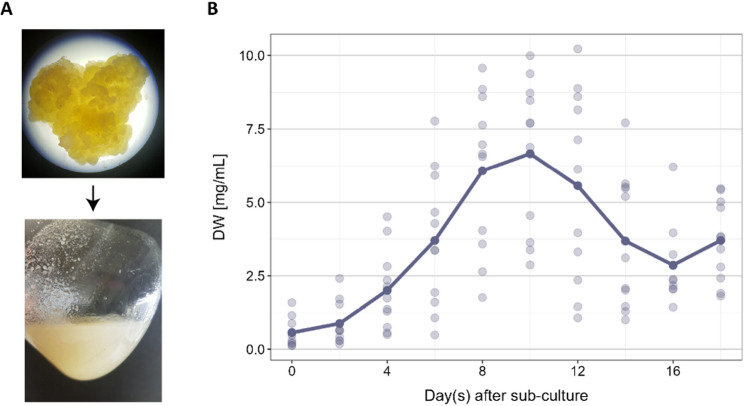
*Ipomoea batatas* cell culture generation and growth. **A** One-month-old callus derived from sweet potato stem (top) and a 7-day-old cell suspension culture (bottom). **B** Growth curve showing changes in dry weight over time; measurements were taken every second day

Starting from sweet potato stems, we first generated callus and subsequently cell suspension cultures (Fig. 2A). Growth parameters are critical when working with cell suspensions. In particular, they are important for identification and choosing the right time for treatments or passaging. There are many methods to characterise the growth of cells and to build a growth curve (fresh or dry biomass, number of cells, packed cell volume, DNA content, etc.) [19]. We chose a biomass sampling for dry weight measurement.

We observed an increase in dry weight during the first 10 days, especially from day 4, which was also supported by the visible rise of culture density (Fig. 2A). This increase indicates the start of the exponential (logarithmic) phase from day 4 to day 8 (Fig. 2B), when cells are actively proliferating and suitable for a treatment analysis. After 10 days, the DW was decreasing steadily, as visually the cell density stopped changing and the colour of the cell suspension changed from white to yellowish with the formation of aggregates, indicating that the cells were undergoing senescence. The 7th day was chosen for all analysis since, as mentioned, cells are metabolically active but yet in their mature stage before senescence. The cells’ viability was confirmed by FDA/PI staining (Additional file 1).

### Oxidative burst

**Fig. 3:**
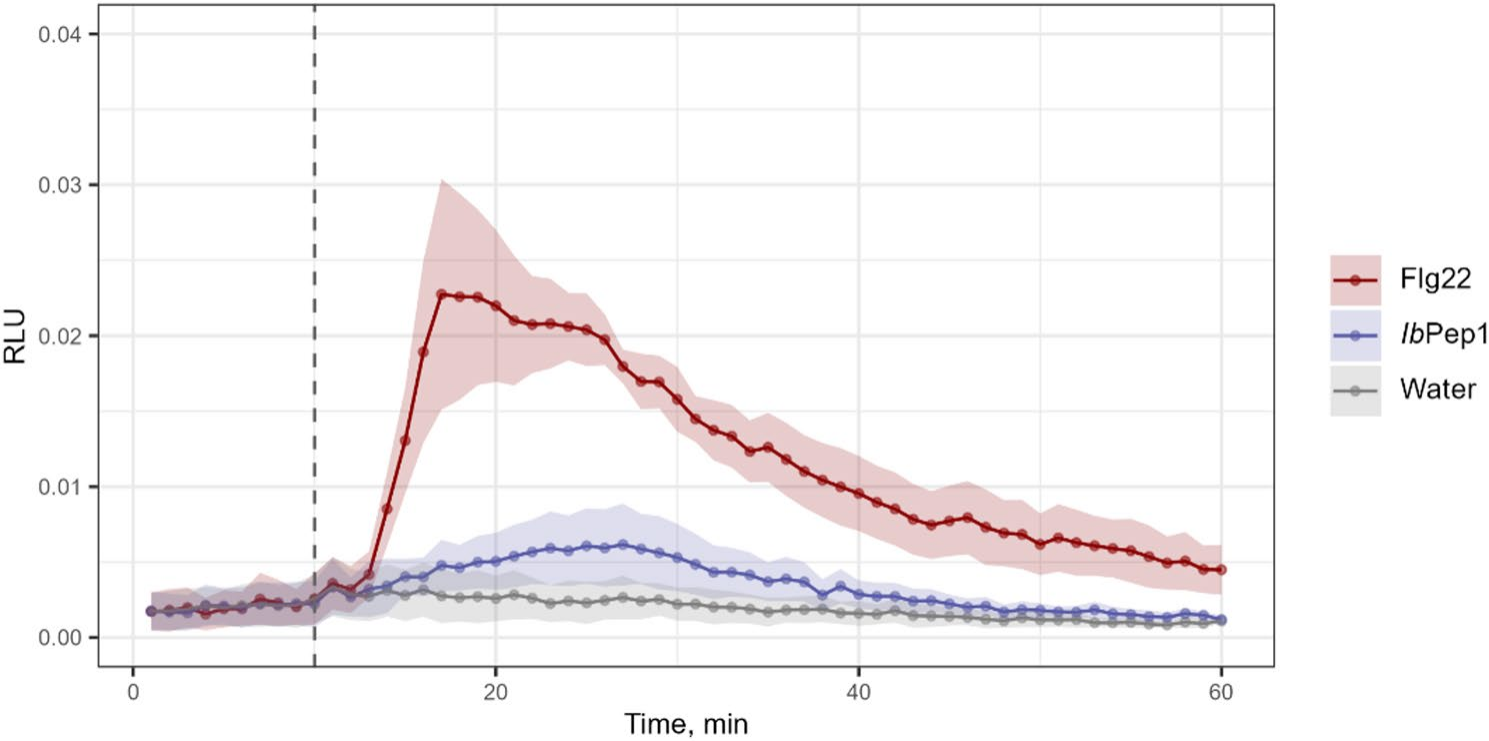
Reactive oxygen species (ROS) dynamics in cell suspensions upon treatment with *Ib*Pep1, flg22 (positive control), or water (negative control). The dashed vertical line indicates the time of the treatment. Shaded areas represent the standard error of the mean (SEM). The experiment was conducted 3 times, each dot representing the mean of 5 technical replicates. A linear mixed-effects model was used to analyse treatment effects over time (fixed effects) with an independent repetition as a random effect. The Benjamini-Hochberg procedure was applied for false discovery rate (FDR) correction. Statistical results are provided in the Additional file 2

In stressed plants, reactive oxygen species (ROS) are among the earliest signals in plants, initiating numerous downstream defence processes in response to both abiotic and biotic stimuli. In this study, we used the luminescent probe L-012 to test whether *Ib*Pep1 can stimulate ROS production in *I. batatas* cell cultures. Flg22 was used as a positive control, and water as a negative control. We observed a clear increase in relative luminescence units (RLU) after flg22 treatment and a smaller rise after *Ib*Pep1 application (Fig. 3). However, statistical analysis revealed that a significant increase compared with the control was detected only in flg22-treated cells, between 15 and 42 min after treatment (Additional file 2).

### Stress phytohormones

**Fig. 4:**
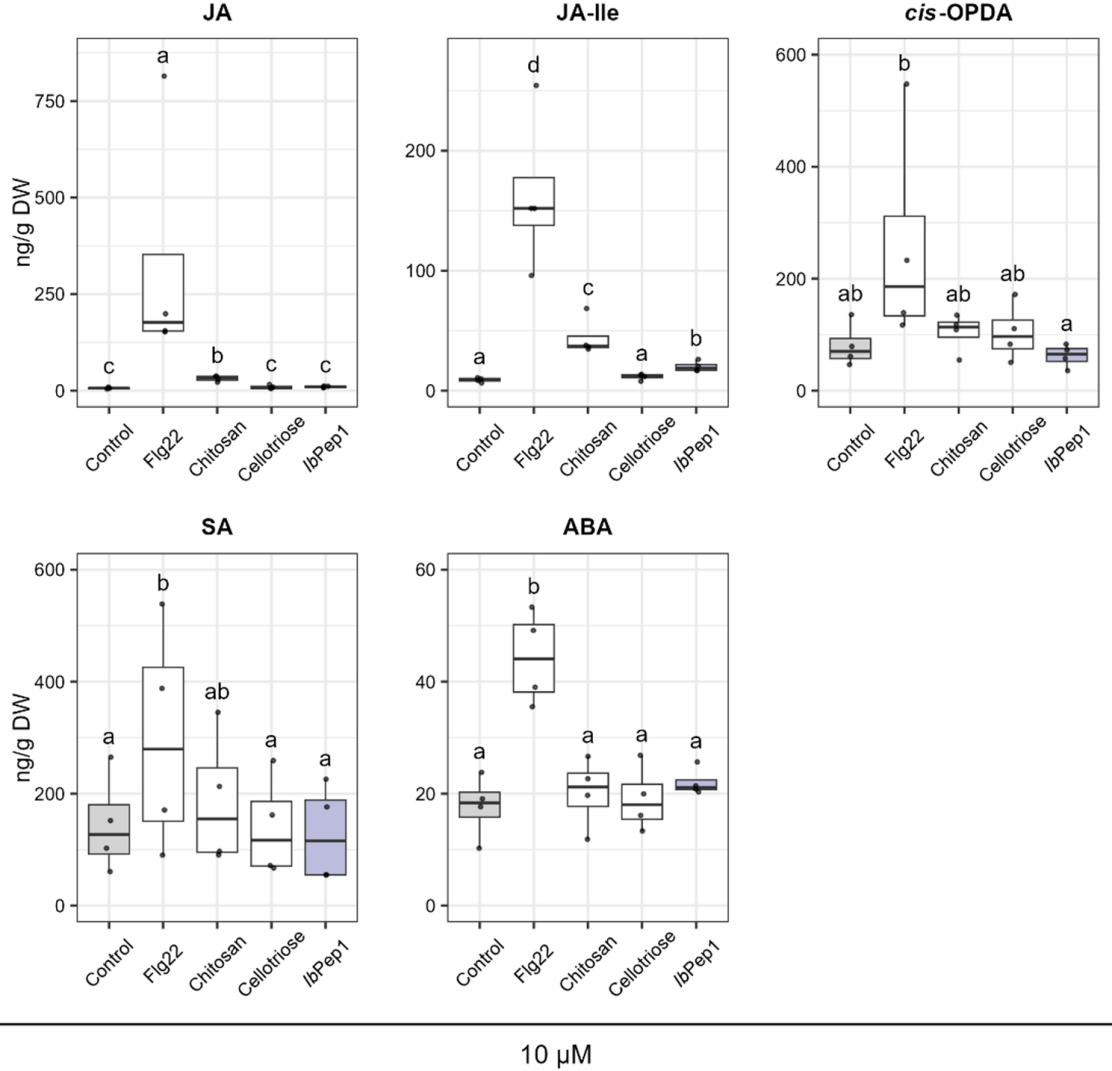
Changes in major stress-related phytohormones in response to different elicitor treatments (10 µM). Each phytohormone was analysed using a linear mixed-effects model with treatment as a fixed effect and block as a random effect to account for batch variation. Pairwise differences among all five treatments were tested using Tukey’s HSD adjustment. Different letters indicate significant differences between treatments (*p* < 0.05; compact letter display). The horizontal line represents the median, data points beyond the whiskers represent outliers (values outside of 1.5 × interquartile range), *n* = 4

It has been proven that different MAMPs and DAMPs influence plant defence responses via phytohormones. We used *Ib*Pep1 to test whether it affects the latter and to compared it, in parallel, with known elicitors. After treating *I. batatas* cell cultures with different elicitors at final concentrations of 1 or 10 µM, we analysed the accumulation of JA, JA-Ile, *cis*-OPDA, SA, and ABA two hours post-treatment. As the phytohormone assessment was designed as a block experiment, we first checked the significance of our treatment (fixed effect) before proceeding with the statistical analysis, which revealed significance across all models. Tukey-adjusted pairwise comparisons revealed that at a concentration of 1 µM, flg22 was the only elicitor that significantly contributed to an increase in all measured phytohormones (Additional file 3). At a concentration of 10 µM, flg22 again influenced phytohormone concentrations, while chitosan had a significant effect only on JA and JA-Ile. Interestingly, we found that also *Ib*Pep1 increased the level of JA-Ile in the treated cells, albeit to a lesser extent (Fig. 4). Neither concentration of cellotriose influenced the phytohormones.

### Free amino acids

**Fig. 5:**
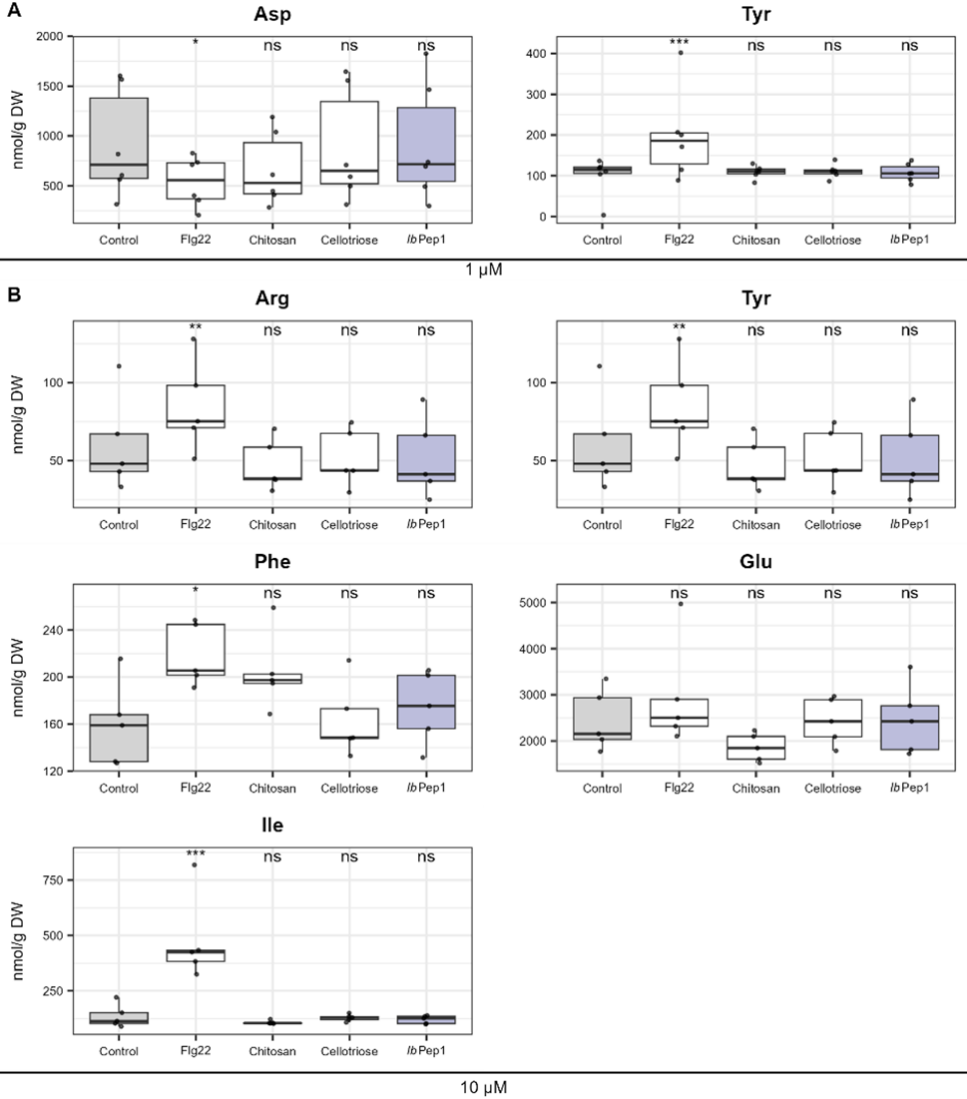
Free amino acids affected by elicitor treatments. Each amino acid was analysed using a linear mixed-effects model with treatment as a fixed effect and block as a random and compared to a null model including only the random effect. Treatment effects were evaluated using Dunnett-type treatment-vs-control contrasts. **A** Amino acids significantly affected by elicitor treatments at 1 µM. **B** Amino acids significantly affected by elicitor treatments at 10 µM. Asterisks indicate significant differences from the control group (*n* = 6 for 1 µM; *n* = 5 for 10 µM): *p* < 0.05 (*), *p* < 0.01 (**), *p* < 0.001 (**)

Amino acids are primary metabolites involved in diverse plant immunity mechanisms and metabolism reprogramming [32]. As with the phytohormone analysis, we assessed their response to common DAMPs and MAMPs, and compared these effects to the signaling peptide *Ib*Pep1. Among the 18 measured amino acids, we pre-selected those showing a significant treatment effect (*p* < 0.05) based on mixed-effect model comparisons. Each amino acid was tested using a linear mixed-effects model with treatment as a fixed effect and block as a random effect. With Dunnett contrasts against the control group, we discovered a significant effect only upon flg22 treatment: for aspartate and tyrosine at 1 µM (Fig. 5A), and for tyrosine, arginine, phenylalanine, and isoleucine at a concentration of 10 µM (Fig. 5B). *Ib*Pep1 treatment didn’t significantly (*p* < 0.05) affect amino acid levels at measured concentrations compared to the control.

### Untargeted metabolomics

**Fig. 6:**
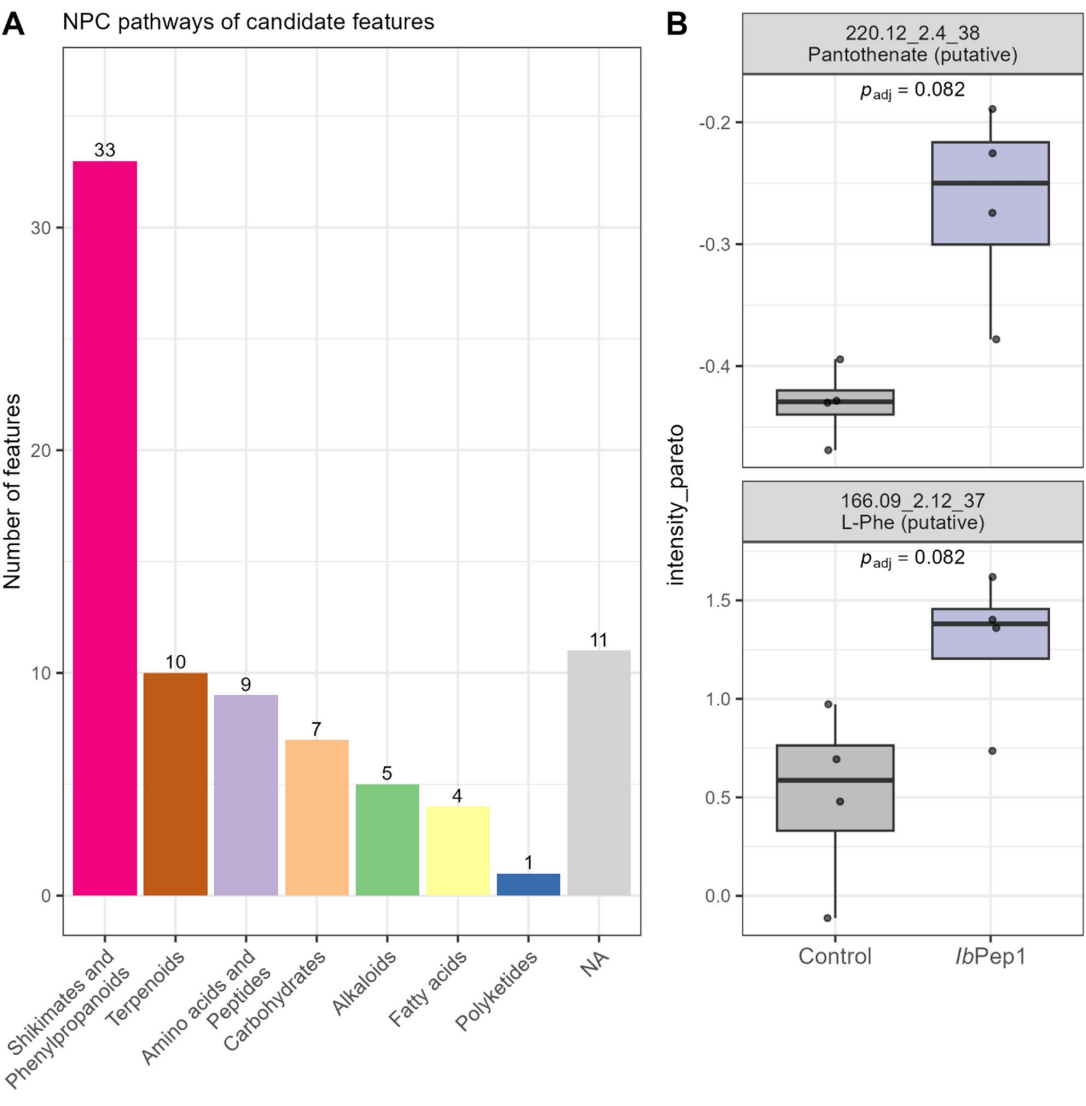
Untargeted metabolomics of *I. batatas* cells after 24 h of treatment. **A** CANOPUS-assigned NPC pathways of all VIP features (VIP score > 1). **B** Relative abundances of two high scored VIP features with a putative compound annotation by CSI: FingerID. Feature names are composed of m/z, retention time, and feature ID. *p*-values correspond to false discovery rate (FDR)-adjusted *p*-values from Wilcoxon rank-sum tests

In order to test the global metabolic changes in the cell suspension upon *Ib*Pep1 treatment, we further used cells harvested after 24 h of volatile collection for untargeted metabolomics. A PLS-DA model was built to explore *Ib*Pep1-treatment-related differences in an untargeted metabolomics dataset comprising *n* = 8 samples (4 vs. 4). Cross-validation indicated moderate predictive performance (Q² ≈ 0.424). However, permutation testing using the R-package “ropls” (1000 permutations) showed that the model did not reach statistical significance (pQ²≈0.177; pR²Y ≈ 0.241), indicating a risk of overfitting. Given the lack of permutation significance and the very small sample size, the PLS-DA model is treated as exploratory and not as a validated classifier. Therefore, variable importance in projection (VIP) scores are used only for feature prioritization and hypothesis generation, not as evidence of statistically significant group discrimination. After identifying the features with the highest VIP scores and linking them to annotated pathways/classes annotated in CANOPUS by NPClassifier, we found that most VIP features (81 total; 12 unassigned) were associated with seven metabolic pathways (Fig. 6A). The most affected were “Shikimates and Phenylpropanoids,” “Terpenoids,” and “Amino Acids and Peptides” (FIG). Features in “Shikimates and Phenylpropanoids” were mainly annotated by a class “Cinnamic acids and derivatives” (Additional file 5). Among the top-ranked features, two metabolites were annotated with high confidence using CSI: FingerID (Fig. 6B, Additional file 5) and were putatively assigned as pantothenate and L-phenylalanine. Both metabolites showed higher relative intensities in the *Ib*Pep1 treatment compared with the control; however, these differences did not remain significant after multiple-testing correction (Fig. 6B).

### Volatile emission

**Fig. 7:**
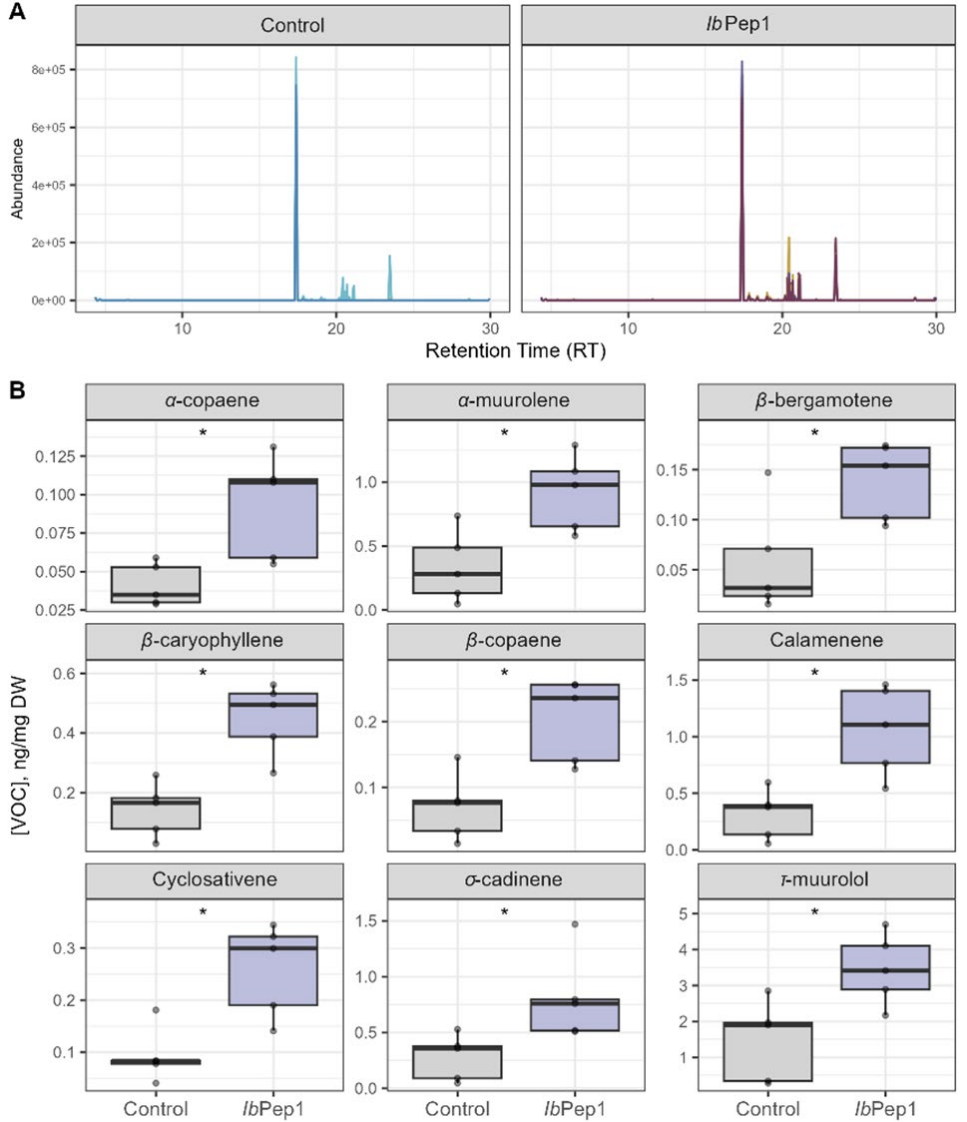
Volatile profile and volatiles affected by*Ib*Pep1 treatment. **A** Representative chromatograms from each treatment demonstrate the presence only of sesquiterpenes in the profile. **B** Identified VOCs which showed significant differences upon *Ib*Pep1 treatment. Box plots show *n* = 4 biological replicates, with the line representing the mean. Asterisks indicate *p* < 0.05 (adjusted using the Benjamini-Hochberg FDR method) from a Student’s t-test or Wilcoxon rank sum test (if data were not normally distributed). Results are reported in the Additional file 7

Plant volatiles are organic compounds that are emitted through plant membranes, trichomes, osmophores, and stomata. Various volatile molecules have been found to regulate intra- and interplant communication and defend plants against biotic and abiotic stressors [33]. To investigate whether *Ib*Pep1 influences volatile emission in plant cell suspensions, we treated cells with the peptide and water (as a control), collected the compounds and analysed them by GC-based methods in combination with databases. During our measurements, the volatile profile of *I. batatas* cell suspensions consisted only of sesquiterpenes and one sesquiterpenoid (Fig. 7A). The following peaks were identified: β-caryophyllene, α-copaene, σ-cadinene, calamenene, β-bergamotene, β-copaene, α-muurolene, cyclosativene, γ-muurolene, eremophylene, and τ-muurolol (a sesquiterpenoid). The last two were present in the highest amount. Treatment with *Ib*Pep1 positively affected volatile emission; a significant effect (*p* < 0.05) was detected for α-copaene, α-muurolene, β-bergamotene, β-caryophyllene, β-copaene, calamenene, cyclosativene, σ-cadinene, and τ-muurolol (Fig. 7B). The only exception was the increase in the sesquiterpene γ-muurolene and the highly abundant eremophylene, where no significant difference was found (*p* = 0.17 and *p* = 0.23, respectively, Additional file 6, 7).

## Discussion

Plant cell suspension cultures depict a valuable and versatile model system to investigate plant metabolism, signaling, and stress responses under directed and controlled conditions. Unlike whole-plant systems, cell cultures offer high reproducibility and allow precise manipulation of environmental or chemical factors, making them especially useful for dissecting molecular mechanisms of plant defence and metabolism. Moreover, they provide a platform for rapid testing of bioactive compounds before validating them in intact plants. In this study, we set up and characterised an *I. batatas* cell suspension culture as a controllable system for studying plant defence responses. We confirmed its metabolic reactivity using the well-known MAMP flg22, which demonstrated that the culture retains functional signaling capacity. Such systems provide a simplified yet physiologically relevant platform for studying both early and late defence responses, offering an efficient alternative to experiments involving whole plants. Using this model, we further investigated the role of the recently identified signaling peptide *Ib*Pep1 in regulating metabolic and defence-related processes in sweet potato.

### ROS signaling

Reactive oxygen species are considered to be a double-edged sword of life: despite their toxic nature, they are beneficial for the organisms’ development, adaptation, and survivability. It is important for organisms to maintain ROS at the basal level because of its oxidative properties. Therefore, organisms developed systems to regulate ROS levels to prevent molecules from damage and maintain their signaling role [34]. One of the most used assays to detect and measure ROS ones is the luminol-based assays, which use chemiluminescence for ROS detection. We used luminol-derived agent L-012 to analyse ROS changes for testing the response activity of the cell culture. As a typical elicitor, we applied a bacteria-derived peptide flg22 and used water treatment as a control. Our results confirmed published studies showing that the bacterial peptide can promote ROS production fast in plant cell suspensions [20, 35, 36]. Regarding plant peptides, the available data is variable, showing that the plant peptides may both amplify the ongoing defence processes as well as regulate them to avoid excessive responses [37]. *At*Pep1 was shown to activate ROS signaling in the root [38] and even enhanced it by MAMPs pre-treatment [39]. In contrast, *Zea mays* immune signaling peptide Zip1 did not induce an oxidative burst in leaf discs [40]. Moreover, Arabidopsis endogenous peptide RAPID ALKALINIZATION FACTOR 23 (RALF23) inhibited ROS production induced by the bacterial peptide elf18 [41]. Together, these observations underscore the complexity of peptide signaling pathways and the importance of continued research to elucidate their roles in plant defence.

### Stress phytohormones

The multifunctionality of phytohormones and their control over physiological processes make research into them essential. The mode of action of most phytohormones is well characterised; for example, JA-Ile is known to accumulate rapidly in response to various stimuli, including herbivory, wounding, or certain microbial signals. Therefore, we investigated the impact of the sweet potato signaling peptide *Ib*Pep1 on plant defence by measuring the levels of several stress-related phytohormones. Microbe- and damage-associated molecular patterns were also included for comparison.

Consistent with previous studies [42, 43], we detected the positive effect of flg22 at both 1 and 10 µM on JA/JA-Ile level, but also on ABA and SA (Fig. 4, Additional file 3). Chitosan effect on jasmonates, JA and JA-Ile, was observed only at the higher concentration. We didn’t detect any effect of cellotriose, even though it has been shown that high levels of DAMPs expression, specifically oligogalacturonides, stimulated SA production in Arabidopsis plants [44]. Regarding *Ib*Pep1 treatment, we observed an increase in the level of JA-Ile upon treatment with a 10 µM concentration (Fig. 4). A similar result was documented by Lu et al. [15], where applied *Ib*HypSysIV slightly increased the level of JA-Ile in sweet potato leaves. Earlier study revealed that *Ib*HypSysIV upregulated expression of two genes involved in the jasmonate biosynthesis (*Ib*-13-LOX and *Ib*AOS) [14]. The interaction between peptide and non-peptide hormone signaling pathways is well studied in animals. Despite being repeatedly observed in plants over the last few decades, the link between them remains unclear [45, 46].

### Free amino acids

Amino acids are crucial components not only of primary metabolism but also of natural product biosynthesis. Prominent examples include the aromatic amino acids tryptophan, tyrosine, and phenylalanine, which are synthesized from chorismate and serve as precursors for many defensive compounds in plants [47, 48]. In our amino acid analysis, we observed that flg22 affected the levels of tyrosine, aspartate, arginine, phenylalanine, and isoleucine (Fig. 5). Aromatic tyrosine and phenylalanine are involved in the synthesis of phenylpropanoids [49, 50]. Phenylpropanoids encompass a wide spectrum of compounds, ranging from large lignin polymers to simple phenolics that enhance plant resistance to biotic and abiotic stresses [51, 52]. Tyrosine also serves as a precursor for cyanogenic glycosides and isoquinoline alkaloids [49]. Isoleucine is a component of the bioactive form of jasmonates, JA-Ile, which is produced through the conjugation of JA with isoleucine by the JA-amino acid synthetase JAR1 [53]. Arginine, with the highest N: C ratio among amino acids, can function as a major organic nitrogen source and transport form [54]. Notably, arginine has also been implicated in nitric oxide (NO) biosynthesis, a small lipophilic molecule involved in plant development and defence [55]. The only amino acid that decreased in flg22-treated cells compared with the control was aspartate (Fig. 5A). In a study where the aspartate-glutamate carrier 1 enzyme was knocked down, the resulting decrease in aspartate inhibited cell proliferation [56], suggesting that aspartate may be a limiting factor for cell growth. Conversely, several studies have reported an elevation of aspartate levels upon fungal infection [57]. Although *Ib*Pep1 did not elicit measurable changes in amino acid levels, the pronounced response to flg22 highlights the sensitivity of plant cells to microbial peptide elicitors.

### Untargeted metabolomics changes

We further investigated the effect of the signaling peptide *Ib*Pep1 on the metabolism of sweet potato cells. A 24-hour treatment was applied to capture global metabolic changes. The most pronounced alterations were observed in the shikimate and phenylpropanoid pathway, followed by terpenoids and amino acid-related compounds. Within the first group, 19 features were putatively identified as cinnamic acids and derivatives. This finding is consistent with previous reports showing that the signaling peptide *Ib*HypSys regulates phenylpropanoid pathway genes in both positive and negative directions [14]. In that study, it was also shown that wounded leaves and those treated with the peptide accumulated lignins, which provide mechanical stability to the cell wall [52]. Lignin is composed of simple phenolic compounds, including cinnamic acids, benzoic acids, and their derivatives, which may explain the observed accumulation of some cinnamic acid derivatives. Beyond their structural role, cinnamic acids have been reported to regulate photosynthesis [52] and contribute to plant defence against biotic stressors [58]. High percentage of features assigned to “Terpenoids” in our study are consistent with the volatile emission results. Similarly, *Zm*Pep3 has been shown to upregulate terpene synthase gene expression in maize (*Zea mays*), further supporting the role of peptide signaling in terpenoid-mediated plant defence [9]. Previously, Narváez-Vásquez and Ryan [59] demonstrated that the expression of the systemin precursor gene influenced the total number of proteins, including defensive proteins in leaves, as well as storage protein levels in potato tubers and the total amount of free amino acids. Although no significant changes were detected in the measured amino acids after two hours of treatment, analysis of high-scored features from the exploratory PLS-DA revealed one of the top-10 VIP features to be putatively annotated as L-Phe. Univariate analysis of its relative abundance indicated an increase upon *Ib*Pep1 treatment, although this effect was not statistically significant (*p*_adj_=0.082). This may indicate that phenylalanine serves as a precursor for the biosynthesis of phenolic compounds, which may be involved in lignin formation [52]. The feature with the highest VIP score was putatively annotated as pantothenate (vitamin B5) and showed also a non-significant increase (*p*_adj_=0.082) upon the peptide treatment. Pantothenate is synthesised by plants and functions as a cofactor in lipid metabolism, including fatty acid synthesis and pyruvate decarboxylation, as well as in specialised metabolite biosynthesis such as lignin formation [60]. Taken together, these observations suggest that *Ib*Pep1 may influence multiple metabolic routes, including phenylpropanoid, terpenoid and amino acid-related pathways, and may indicate a broader metabolic adjustment in response to peptide signalling.

### VOCs emission

As mentioned in the methods, we cultivated sweet potato cell culture in the dark in a medium supplemented with sucrose and growth promoters. These conditions are shown to inhibit photosynthetic activity of plastids and potentially even lead to their functional loss [61]. This fact drastically affects the number of biosynthetic pathways, including volatile organic compounds, in particular, terpenoids. While synthesis of monoterpenes and diterpenes requires geranyl pyrophosphate and geranylgeranyl pyrophosphate, which are synthesised in plastids, the sesquiterpene precursor farnesyl pyrophosphate, in contrast, is synthesised in the cytosol.

Almost all discovered sesquiterpenes in our study were increased upon peptide treatment (Fig. 7B). α-copaene, β-copaene, and β-caryophyllene were already confirmed to be increased upon mechanical damage and *Spodoptera littoralis* feeding in sweet potato cultivars [62], which can potentially confirm the defence-stimulating role of *Ib*Pep1 peptide. β-caryophyllene, as one of the most widespread floral scent compounds, has been proven to attract pollinators and parasitic wasps and to play a role in defence against bacterial pathogens [63]. Calamenene was previously found in *Cupressus sp.* [64], but there is no evidence of its stress response role according to our knowledge. The study of Mayanglambam et al. [65] reported the insecticidal and antifeedant roles of the essential oils containing γ-muurolene as one of the main components, but there was no study found in this regard about α-muurolene. The level of high abundant volatiles in *I. batatas* cell culture, eremophylene and γ-muurolene, were not significantly different upon the peptide treatment (Additional file 6, 7).

These results suggest that *Ib*Pep1 may play a role in regulating systemic responses in plants, including the emission of volatiles. Previously, *Ib*HypSysIV was shown to induce the emission of the homoterpene DMNT, whereas *Ib*Pep1 had no significant effect [15]. Similarly, treatment with the elicitor peptide *Zm*Pep3 stimulated the emission of sesquiterpenes in maize [9]. Together, these findings highlight the involvement of plant signaling peptides in indirect anti-herbivory defence through the activation of volatile organic compound biosynthesis. Our results further expand this understanding by suggesting that *Ib*Pep1 may contribute to terpenoid-based defence signaling in sweet potato cells.

## Conclusions

In this study, we investigated the role of *Ib*Pep1, the first elicitor peptide identified in sweet potato, in plant metabolism and defence. We analysed both early and late signaling events. We found that *Ib*Pep1 shares several characteristics with the known *Ib*HypSys peptide, as well as with Peps from other plant families than Convolvulaceae. Although we did not observe any significant effects on ROS production or free amino acid levels, we found that *Ib*Pep1 stimulated the emission of sesquiterpenes, increased the concentration of JA-Ile, and had a strong effect on the phenylpropanoid and shikimate pathways. These results suggest that *Ib*Pep1 may be involved in regulating phenolic compounds and terpenoid biosynthesis, which could inform future research and potential biotechnological applications.

## Supplementary Information

Below is the link to the electronic supplementary material.

Additional File 1. Cells were stained with FDA (green, live) and PI (yellow, dead).

Additional File 2. The table reports pairwise contrasts across time points including the contrast identifier, time point, estimated effect, degrees of freedom, t-ratio, and p-value.

Additional File 3. Each phytohormone was analysed using a linear mixed-effects model with treatment as a fixed effect and block as a random effect to account for batch variation. P-values were adjusted using the Tukey method for multiple comparisons among five treatments. Different letters indicate statistically significant differences among treatments (p < 0.05). The horizontal line represents the median, and crosses indicate means, n = 4. Data points beyond the whiskers represent outliers (values > 1.5 × interquartile range).

Additional File 4. (A) PLS-DA plot showing the separation of the two groups along the first two components. (B) Relative abundances of high scored VIP features, p -values are FDR-adjusted from a Wilcoxon rank sum test.

Additional File 5. All VIP features (VIP score > 1) are listed alongside their VIP score, loading values (Component 1) and the pathway and class assigned by CANOPUS. For features with ID = 37 and 38 putative CSI:FingerID compound annotation.

Additional File 6. Box plots show n = 4 biological replicates, with the line representing the mean. Asterisks indicate p < 0.05 (FDR adjusted) from a Student t-test or Wilcoxon rank sum test (if data are not normally distributed).

Additional File 7. Results of the Shapiro–Wilk test (p_shapiro_g1, p_shapiro_g2), the type of statistical test applied, the p-values, and the adjusted p-values.

## Data Availability

The datasets used and/or analysed during the current study are available from the corresponding author on reasonable request.

## Abbreviations

ABA: Abscisic acid
cis-OPDA: Cis-12-Oxo-phytodienoic acid
DAMPs: Damage-associated molecular patterns
DW: Dry weight
HypSys: Hydroxyproline-rich systemins
JA: Jasmonic acid
JA-Ile: Jasmonoyl-isoleucine
MAMPs: Microbe-associated molecular patterns
PCSs: Plant cell suspensions
Peps: Plant elicitor peptides
RLUs: Relative luminescence units
ROS: Reactive oxygen species
SA: Salicylic acid
VIP features: Variable importance in projection features
VOCs: Volatile organic compounds
