# Supplementary figures and images for: Metabolomic and volatile profiling reveals defence-related effects of IbPep1 in sweet potato cell culture
Source: BMC Plant Biol. 2026 Mar 12;26:636. doi: 10.1186/s12870-026-08547-1 (PMC13063631; doi:10.1186/s12870-026-08547-1)

A

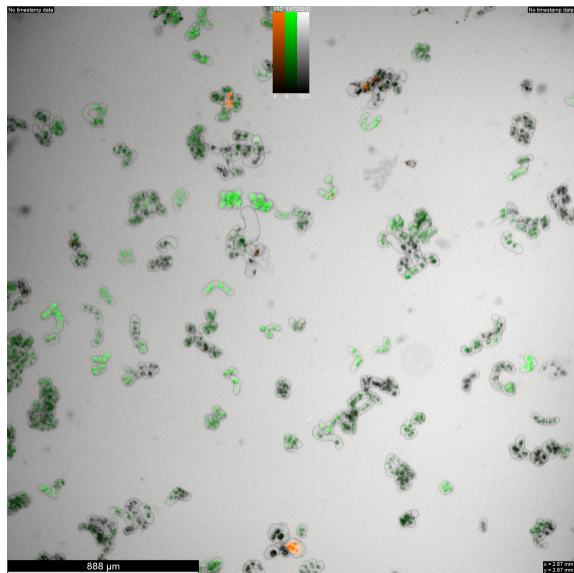

B

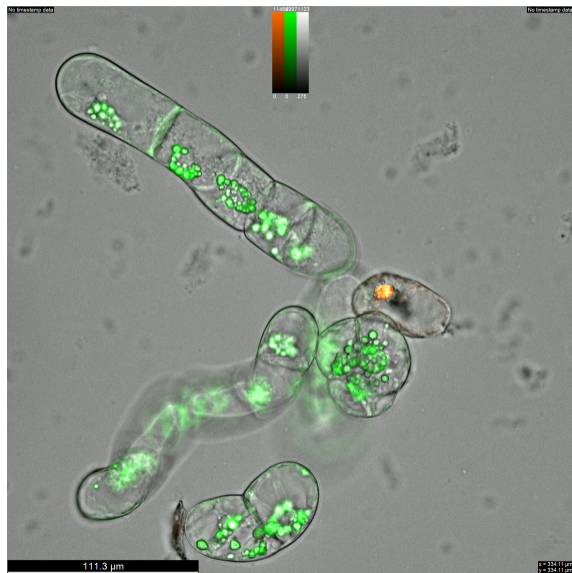

Supplement: Supplementary file 1 — Additional File 1. Cells were stained with FDA (green, live) and PI (yellow, dead). [file 12870_2026_8547_MOESM1_ESM.pdf]

**JA**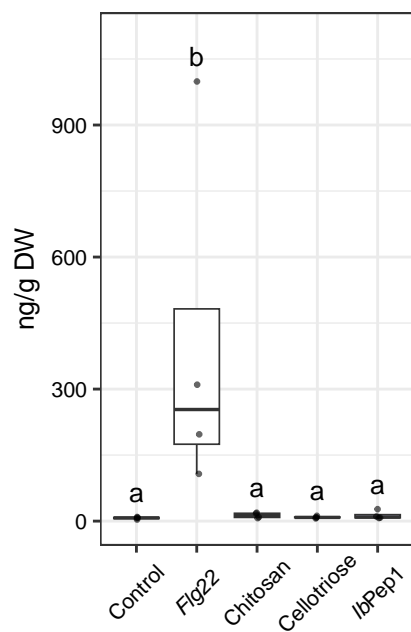**JA-Ile**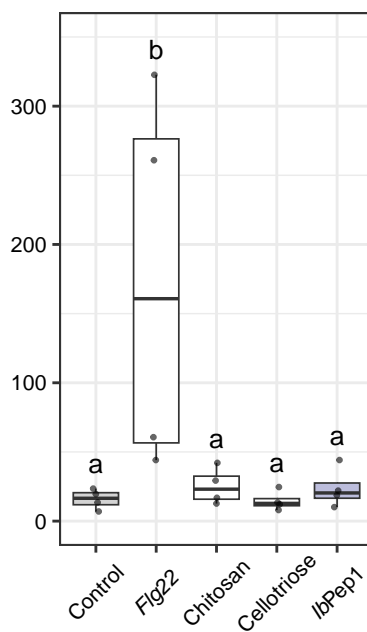***cis*-OPDA**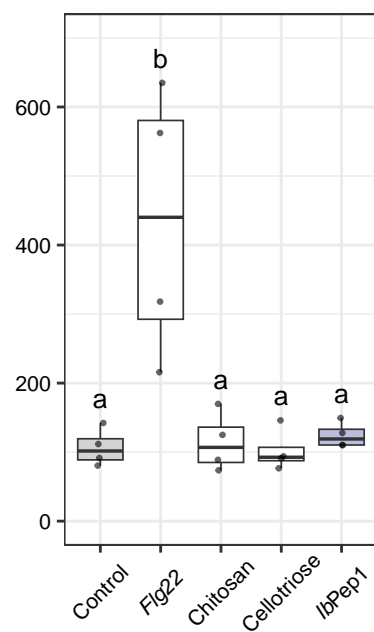**SA**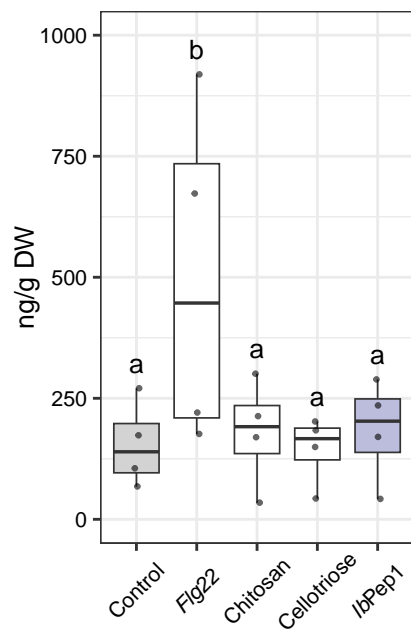**ABA**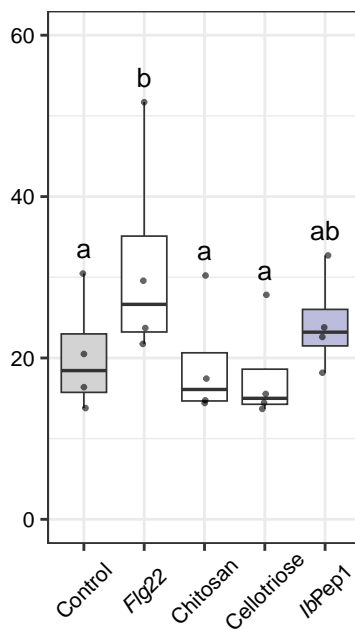

1  $\mu$ M

Supplement: Supplementary file 3 — Additional File 3. Each phytohormone was analysed using a linear mixed-effects model with treatment as a fixed effect and block as a random effect to account for batch variation. P-values were adjusted using the Tukey method for multiple comparisons among five treatments. Different letters indicate statistically significant differences among treatments (p < 0.05). The horizontal line represents the median, and crosses indicate means, n = 4. Data points beyond the whiskers represent outliers (values > 1.5 × interquartile range). [file 12870_2026_8547_MOESM3_ESM.pdf]

**A**      ■ Control    ■ *lbPep1*

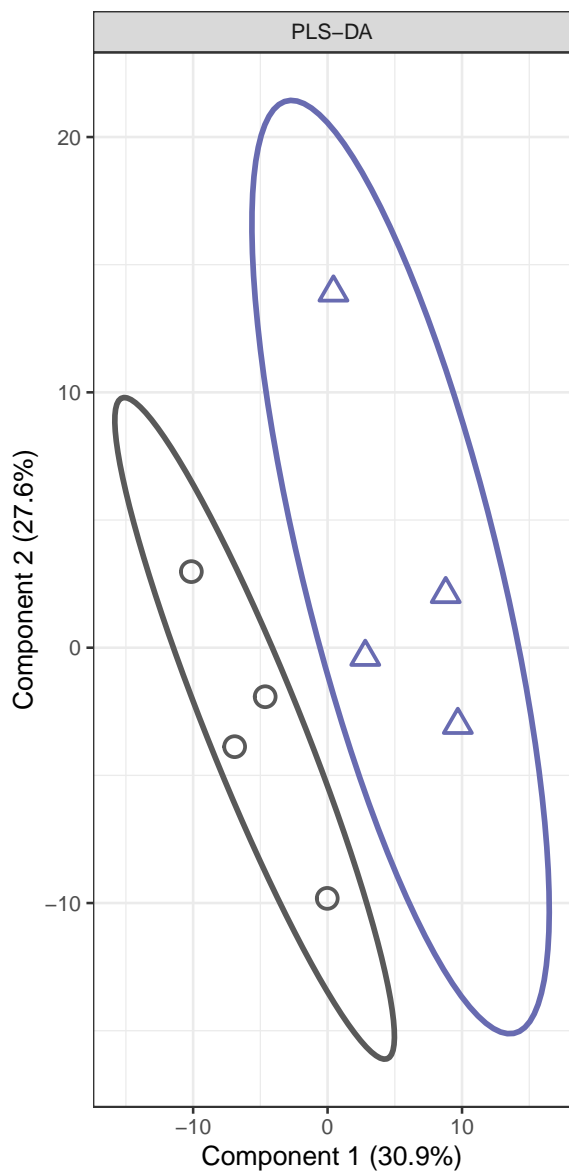

**B**

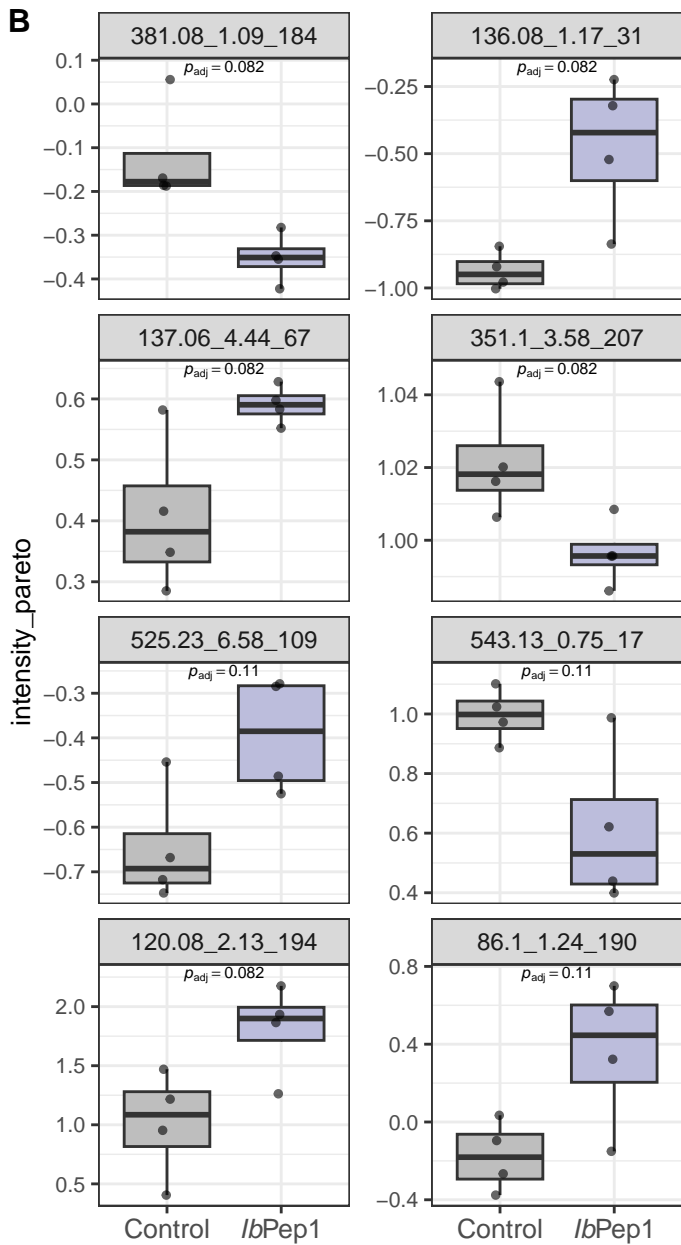

Supplement: Supplementary file 4 — Additional File 4. (A) PLS-DA plot showing the separation of the two groups along the first two components. (B) Relative abundances of high scored VIP features, p -values are FDR-adjusted from a Wilcoxon rank sum test. [file 12870_2026_8547_MOESM4_ESM.pdf]

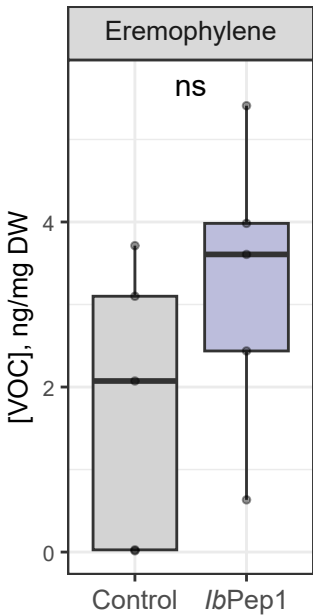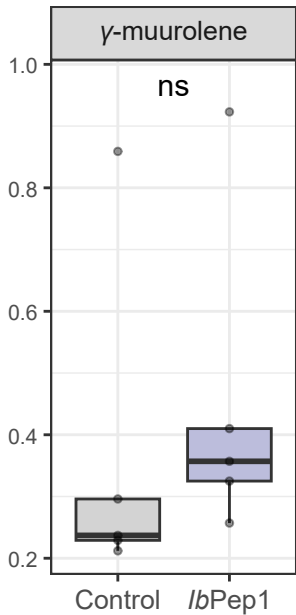

Supplement: Supplementary file 6 — Additional File 6. Box plots show n = 4 biological replicates, with the line representing the mean. Asterisks indicate p < 0.05 (FDR adjusted) from a Student t-test or Wilcoxon rank sum test (if data are not normally distributed). [file 12870_2026_8547_MOESM6_ESM.pdf]
